# Variations in catastrophic health expenditure across the states of India: 2004 to 2014

**DOI:** 10.1371/journal.pone.0205510

**Published:** 2018-10-22

**Authors:** Anamika Pandey, G. Anil Kumar, Rakhi Dandona, Lalit Dandona

**Affiliations:** 1 Public Health Foundation of India, National Capital Region, Gurugram, India; 2 Institute for Health Metrics and Evaluation, University of Washington, Seattle, WA, United States of America; ESIC Medical College & PGIMSR, INDIA

## Abstract

**Background:**

Financial protection is a key dimension of universal health coverage. Catastrophic health expenditure (CHE) has increased in India over time. The overall figures mask the subnational heterogeneity crucial for designing insurance coverage for 1.3 billion population across India. We estimated CHE in every state of India and the changes over a decade.

**Methods:**

We used National Sample Survey data on health care utilisation in 2004 and 2014. The states were placed in four groups based on epidemiological transition level (ETL), defined on the basis of ratio of disability-adjusted life-years from communicable diseases to those from non-communicable diseases and injuries combined, with a low ratio denoting high ETL state group. CHE was defined as the proportion of households that had out-of-pocket payments for health care equalling or exceeding 10% of the household expenditure. We assessed variation in the magnitude and distribution of CHE between ETL state groups and between states of India.

**Results:**

In 2014, CHE was higher in the high (30.3%, 95% confidence interval: 28.5 to 32.1) and higher-middle (27.4%, 26.3 to 28.6) ETL state groups than the low (21.8%, 20.8 to 22.8) and lower-middle (19.0%, 17.1 to 21.0) groups. From 2004 to 2014, CHE increased only in the high and higher-middle ETL groups (1.19 and 1.34 times, respectively). However, the individual states with substantial increase in CHE were spread across all ETL groups. The gap between the highest CHE of an individual state and the lowest was 8-fold in 2014. CHE was disproportionately concentrated among the rich in 2004 for most of India, but in 2014 CHE was distributed equally among the rich and poor because of the substantial increase in CHE among the poor over time.

**Conclusions:**

Better provision of quality health care should be accompanied by financial protection measures to safeguard the poor from increasing CHE in India. The state-specific CHE trends can provide useful input for the planning of the recently launched National Health Protection Mission such that it meets the requirement of each state.

## Introduction

The United Nations 2030 Agenda for Sustainable Development Goals (SDGs) has emphasized on universal health coverage (UHC) which aims to achieve equity in access to quality essential health services, ensuring that the cost of using services does not put households at risk of financial catastrophe [1, 2]. Financial protection is a key dimension of UHC which is central to the achievement of other health targets under the SDGs 2030 [3]. Incidence of catastrophe health expenditure (CHE) is a measure of the performance of the health system in a country and is a useful indicator to monitor the progress towards UHC [4].

There has been growing commitment in recent years to achieve the goal of UHC. A fundamental concern of governments in striving for UHC is how to finance such a health system. This concern is more relevant for low- and middle-income countries which have low public investment in health. Globally, the share of out-of-pocket (OOP) payments for health care has been declining but the OOP spending as a share of income has not been declining [5]. Worldwide around 800 million people spend more than 10% of their household budget on health care, and almost 100 million people are pushed into extreme poverty each year because of OOP health expenses [4]. In India, the OOP expenditure as share of total health expenditure has declined from 69% in 2004–05 to 64% in 2013–14 [6]. However, the proportion of households with OOP payments equalling or exceeding 10% of their consumption expenditure has increased more than two times during the same time period [7]. In spite of the unprecedented economic growth, the nation suffers from tremendous shortfalls in health care financing and OOP payments as proportion of total health expenditure remains much higher than the global average. Achieving significant reductions in OOP payments, CHE and impoverishments due to health expenditure has been envisaged in the National Health Policy 2017 [8].

India is a federal union comprising of 29 states and 7 union territories which vary widely in terms of their physical and financial resources, health indicators, and disease burden. Just looking at the overall figure may not provide the much needed insight into the specific problem of the states which is fundamental for designing insurance coverage for the 1.3 billion population across India. We assess the variation in CHE across states grouped by the epidemiological transition level and also across individual states over the most recent decade for a more informed policy decision. We divided states on the basis of their epidemiological transition level to capture the variation in CHE across the states with varying pattern of mortality, fertility, life expectancy, causes of death, and population age distribution [9]. Epidemiological transition resulting into higher burden of non-communicable diseases than the communicable diseases reflects not only the changing lifestyle and diet but also population ageing. The findings from this study will be useful in designing a more specific strategies to reduce the inter-state variations in out-of-pocket payments for health care.

## Materials and methods

We used data from two recent nationwide health care surveys done in India by the National Sample Survey Organisation in 2004 and 2014 [10, 11]. Data were collected from a nationally representative sample of 73,868 households with 383,338 persons in NSS 2004 and 65,932 households with 333,104 persons in NSS 2014. These surveys collected information on direct medical and non-medical expenditure on all individuals in the households pertaining to each episode of hospitalisation in the reference period of one year, and each spell of ailment treated as outpatient in 15 days reference period. OOP payments were obtained in both these surveys after the deduction of any amount reimbursed or expected to be reimbursed or paid directly by the employer, insurance companies, or other agencies. We included the expenditure on child birth in the hospital in both the surveys while calculating the hospitalisation expenditure. Any expenditure on the immunisation of children, pre and post-natal care, and child birth (not in the hospital) in the last one year were included in both the surveys while calculating the outpatient care expenditures. Details of the items used to access OOP payments on inpatient and outpatient care are presented in S1 Table.

Our outcome variables were per capita OOP payments for health care in the most recent month, and the occurrence of CHE in the most recent month. There are two definitions of CHE used in the literature to measure the burden of OOP payments for households [12–15]. Under the first, OOP payments are estimated as a proportion of total household expenditure, and under the second it is estimated as the proportion of household’s capacity-to-pay. The threshold for measuring CHE under both these definitions are arbitrary, however 10% of household consumption expenditure, and 40% of household capacity to pay are the most widely used cut-offs [15, 16]. We defined CHE as the proportion of households with OOP payments for health care equalling or exceeding 10% of the household consumption expenditure which is in line with the previous studies [7, 14, 17–19]. Since the information on household consumption expenditure was available in these surveys only in aggregate in the 30 days reference period, we could not use the capacity-to-pay approach for measuring CHE. The expenditure for inpatient and outpatient care were collected for different recall periods, we therefore, converted them into the same recall period of one month to calculate the per capita OOP expenditure and determine the occurrence of CHE in households. To enable comparability across the two surveys, INR costs in 2004 were converted to 2014 constant prices using the gross domestic product deflators [20]. The INR cost at 2014 prices from both the surveys were then converted to United States dollars (US$) using the average 2014 exchange rate (US$ 1 = 63.3 INR) [21].

For analysis, the states of India were grouped according to their epidemiological transition level (ETL) in 2016, which was defined as the ratio of all-age disability-adjusted life-years (DALYs) due to communicable, maternal, neonatal, and nutritional diseases (CMNNDs) versus those due to non-communicable disease (NCDs) and injuries together [22]. DALYs are a summary measure of the health loss burden caused by different conditions, and take into account both premature death and disability in one combined measure [23]. A smaller ETL ratio indicates advancing epidemiological transition–ie, higher burden of NCDs and injuries than CMNNDs. The gradient for classification of states into four ETL groups in 2016 was as follows: low ETL (0.56–0.75), lower-middle ETL (0.41–0.55), higher-middle ETL (0.31–0.40), and high ETL (less than 0.31) [22]. Monthly per capita consumption expenditure (MPCE) adjusted for household size and composition was used as a proxy for economic status. The equivalence scale used was e_h_ = (A_h_+0.5K_h_)^0.75^, where A_h_ was the number of adults in the household and K_h_ was the number of children 0–14 years [7, 24, 25].

The variation in mean per capita OOP payments across states and ETL state groups, and the unadjusted association between CHE and the independent variables derived from survey data are presented as descriptive statistics. We used concentration index to calculate the magnitude of inequality in CHE across all states and ETL state groups in India [26]. Concentration index was computed as twice the weighted covariance of the households with CHE and household’s relative rank in terms of economic status (adult equivalent MPCE is the rank variable in this study) divided by the mean of households with CHE:

$$C=\frac{2}{\mu }Cov_{w\;(y_{i,\;}R_{i})}$$ (1)

Where C stands for concentration index, y_i_ is the binary variable of whether CHE occurred in the i^th^ household, μ is the mean of CHE, and R_i_ is the fractional rank of the i^th^ household in the consumption distribution. The 95% confidence interval (95% CI) for the concentration index was obtained using delta method [27, 28]. The concentration index lies in the interval [−1, 1] [29, 30]. Its positive value indicates that a variable is more concentrated among the advantaged, and vice versa. As de-identified data available in the public domain were utilized, no ethics approval was necessary.

## Results

The highest proportion of households belonged to the low ETL state groups (43.6%) followed by higher-middle (35.8%), high (14.3%) and lower-middle (6.3%) ETL groups in 2014. In 2004, this proportion was 41.5%, 37.0%, 14.9% and 6.6%. High ETL state group had a higher proportion of households with OOP payments than the low ETL group; this differential was 1.18 times in 2004 and 1.43 times in 2014 (Table 1). From 2004 to 2014, the households with OOP payments increased significantly by 1.17 times in the higher-middle and 1.20 times in the high ETL group, but remained similar in the low and lower-middle ETL group. Overall, the mean per capita OOP expenditure was 6.6 (6.50 to 6.77) US$ in 2014. This was 62% higher than that in 2004 (4.10, 4.00 to 4.20 US$). From 2004 to 2014, there was similar increase in mean per capita OOP payments in the three ETL state groups (range: 1.61 to 1.64 times) other than the lower-middle ETL group which had the least increase (1.38 times). The highest OOP payment of an individual state in 2014 was 5.1 times the lowest. From 2004 to 2014, OOP payment increased most in the higher-middle ETL state of Delhi (3.91 times) followed by high ETL state of Goa (2.17 times), low ETL states of Odisha (2.06 times), Chhattisgarh (2.03 times), and Bihar (1.90 times) (Table 1).

**Table 1:** Mean per capita out-of-pocket (OOP) payments for health care by states and ETL state groups in India, NSS 2004 and NSS 2014.

State and ETL groups	Number of households (%)		Households with OOP payments, % (95% CI)		Mean per capita OOP payments (US$) (95% CI)	
	NSS 2004	NSS 2014	NSS 2004	NSS 2014	NSS 2004	NSS 2014
India	73,868	65,932	39.6 (39.0 to 40.1)	43.1 (42.3 to 43.9)	4.10 (4.00 to 4.20)	6.63 (6.50 to 6.77)
**Low ETL state group**	**29,952 (41.5)**	**25,800 (43.6)**	**38.9 (38.1 to 39.7)**	**38.8 (37.5 to 40.0)**	**3.52 (3.37 to 3.66)**	**5.68 (5.46 to 5.89)**
Bihar	4,174 (6.2)	3,167 (7.4)	38.7 (36.5 to 40.9)	38.8 (35.0 to 42.5)	2.71 (2.41 to 3.01)	5.15 (4.76 to 5.54)
Jharkhand	1,996 (2.2)	1,453 (2.5)	33.8 (30.8 to 36.7)	38.0 (32.8 to 43.1)	2.34 (1.97 to 2.71)	4.22 (3.53 to 4.91)
Uttar Pradesh	9,309 (14.2)	7,921 (14.1)	46.1 (44.7 to 47.6)	42.7 (40.5 to 44.9)	3.95 (3.65 to 4.26)	6.21 (5.80 to 6.63)
Rajasthan	3,383 (4.8)	2,912 (5.1)	34.3 (32.0 to 36.6)	36.6 (33.4 to 39.8)	4.40 (4.03 to 4.78)	5.33 (4.54 to 6.12)
Meghalaya	775 (0.2)	832 (0.2)	22.7 (18.6 to 26.7)	27.9 (23.3 to 32.5)	1.48 (1.08 to 1.88)	1.87 (1.59 to 2.14)
Assam	2,630 (2.3)	2,255 (2.6)	36.0 (33.4 to 38.7)	27.2 (23.5 to 30.9)	3.07 (2.77 to 3.36)	4.67 (3.70 to 5.64)
Chhattisgarh	1,470 (2.1)	1,205 (2.2)	31.8 (28.6 to 35.0)	24.5 (19.9 to 29.2)	3.26 (2.55 to 3.97)	6.61 (4.93 to 8.29)
Madhya Pradesh	3,561 (5.7)	3,613 (5.8)	35.8 (33.5 to 38.1)	38.0 (35.0 to 41.1)	3.20 (2.83 to 3.57)	5.43 (4.99 to 5.87)
Odisha	2,654 (3.7)	2,442 (3.8)	32.3 (30.0 to 34.6)	45.4 (41.5 to 49.2)	3.10 (2.82 to 3.37)	6.38 (5.76 to 7.00)
**Lower-middle ETL state group**	**8,959 (6.6)**	**8,858 (6.3)**	**38.1 (36.0 to 40.2)**	**36.5 (33.9 to 39.0)**	**4.04 (3.73 to 4.35)**	**5.56 (5.21 to 5.92)**
Arunachal Pradesh	1,052 (0.1)	626 (0.1)	32.4 (28.0 to 36.9)	45.2 (38.0 to 52.5)	5.18 (3.91 to 6.45)	6.72 (5.71 to 7.72)
Mizoram	1,025 (0.1)	768 (0.1)	17.7 (14.6 to 20.8)	26.6 (22.0 to 31.1)	1.72 (1.21 to 2.22)	2.77 (2.28 to 3.26)
Nagaland	360 (0.1)	576 (0.2)	42.0 (33.7 to 50.2)	21.5 (11.7 to 31.2)	2.87 (1.75 to 4.00)	3.21 (2.85 to 3.57)
Uttrakhand	546 (0.9)	672 (0.8)	35.9 (29.9 to 41.8)	43.3 (35.4 to 51.2)	4.54 (3.47 to 5.60)	5.95 (4.73 to 7.16)
Gujarat	2,806 (4.9)	2,888 (4.6)	38.8 (36.2 to 41.4)	36.7 (33.5 to 39.9)	3.99 (3.48 to 4.49)	5.40 (4.91 to 5.89)
Tripura	1,060 (0.4)	1,408 (0.4)	42.9 (39.0 to 46.8)	31.5 (27.1 to 35.9)	4.70 (3.05 to 6.35)	8.63 (5.89 to 11.37)
Sikkim	520 (0.1)	512 (0.1)	21.0 (16.5 to 25.5)	18.7 (12.9 to 24.5)	2.66 (2.14 to 3.18)	3.56 (2.88 to 4.24)
Manipur	1,590 (0.2)	1,408 (0.2)	35.6 (32.4 to 38.8)	31.0 (27.1 to 34.8)	2.70 (2.33 to 3.06)	5.03 (4.60 to 5.46)
**Higher-middle ETL state group**	**23,859 (37.0)**	**22,262 (35.8)**	**38.0 (37.1 to 38.9)**	**44.6 (43.2 to 46.0)**	**4.42 (4.25 to 4.60)**	**7.24 (7.02 to 7.46)**
Haryana	1,400 (1.8)	1,424 (1.9)	46.8 (43.1 to 50.5)	39.5 (34.3 to 44.7)	5.06 (4.21 to 5.90)	7.25 (6.43 to 8.07)
Delhi	1,039 (1.3)	1,158 (1.2)	21.5 (18.1 to 24.9)	22.2 (18.1 to 26.3)	2.29 (1.50 to 3.08)	8.94 (7.12 to 10.77)
Andhra Pradesh*	5,059 (8.9)	3,942 (8.3)	33.4 (31.5 to 35.3)	48.6 (45.3 to 51.9)	4.93 (4.46 to 5.40)	8.02 (7.45 to 8.59)
Jammu and Kashmir	1,201 (0.7)	1,279 (0.8)	41.9 (37.2 to 46.6)	37.6 (31.7 to 43.4)	3.71 (3.19 to 4.22)	6.37 (5.65 to 7.09)
Karnataka	3,365 (5.0)	2,959 (5.5)	30.7 (28.6 to 32.9)	42.4 (39.0 to 45.9)	3.67 (3.23 to 4.11)	6.53 (6.05 to 7.01)
West Bengal	5,049 (8.5)	5,019 (8.5)	44.9 (42.9 to 46.8)	54.3 (51.3 to 57.2)	3.91 (3.60 to 4.23)	6.67 (6.24 to 7.10)
Maharashtra	5,314 (10.5)	5,403 (9.2)	40.3 (38.3 to 42.2)	37.7 (35.2 to 40.1)	4.92 (4.58 to 5.27)	7.61 (7.14 to 8.08)
Union territories other than Delhi	1,432 (0.3)	1,078 (0.3)	35.0 (30.9 to 39.2)	50.6 (43.3 to 57.9)	4.48 (3.54 to 5.41)	6.51 (5.49 to 7.54)
**High ETL state group**	**11,098 (14.9)**	**9,012 (14.3)**	**45.9 (44.5 to 47.4)**	**55.3 (53.0 to 57.5)**	**5.20 (4.88 to 5.51)**	**8.40 (8.01 to 8.79)**
Himachal Pradesh	1,439 (0.7)	896 (0.6)	39.0 (35.6 to 42.5)	39.2 (33.5 to 45.0)	5.85 (5.09 to 6.62)	6.84 (5.93 to 7.74)
Punjab	1,492 (2.4)	1,529 (2.2)	50.6 (46.8 to 54.5)	57.2 (52.1 to 62.3)	5.49 (4.55 to 6.43)	8.24 (7.23 to 9.25)
Tamil Nadu	5,139 (8.0)	3,917 (8.0)	34.7 (32.8 to 36.7)	47.8 (44.6 to 51.1)	4.87 (4.30 to 5.44)	7.77 (5.81 to 9.72)
Goa	199 (0.2)	192 (0.1)	36.6 (23.1 to 50.0)	71.5 (58.7 to 84.3)	3.64 (2.69 to 4.59)	7.88 (7.34 to 8.43)
Kerala	2,829 (3.6)	2,478 (3.3)	69.4 (67.2 to 71.6)	74.6 (71.5 to 77.7)	5.35 (4.92 to 5.77)	9.43 (8.64 to 10.23)

NSS = National Sample Survey; ETL = Epidemiological transition level; CI = Confidence interval; US$ = United States Dollars

*For comparison purpose Andhra Pradesh has been considered as an undivided state in NSS 2014.

**Fig 1:**
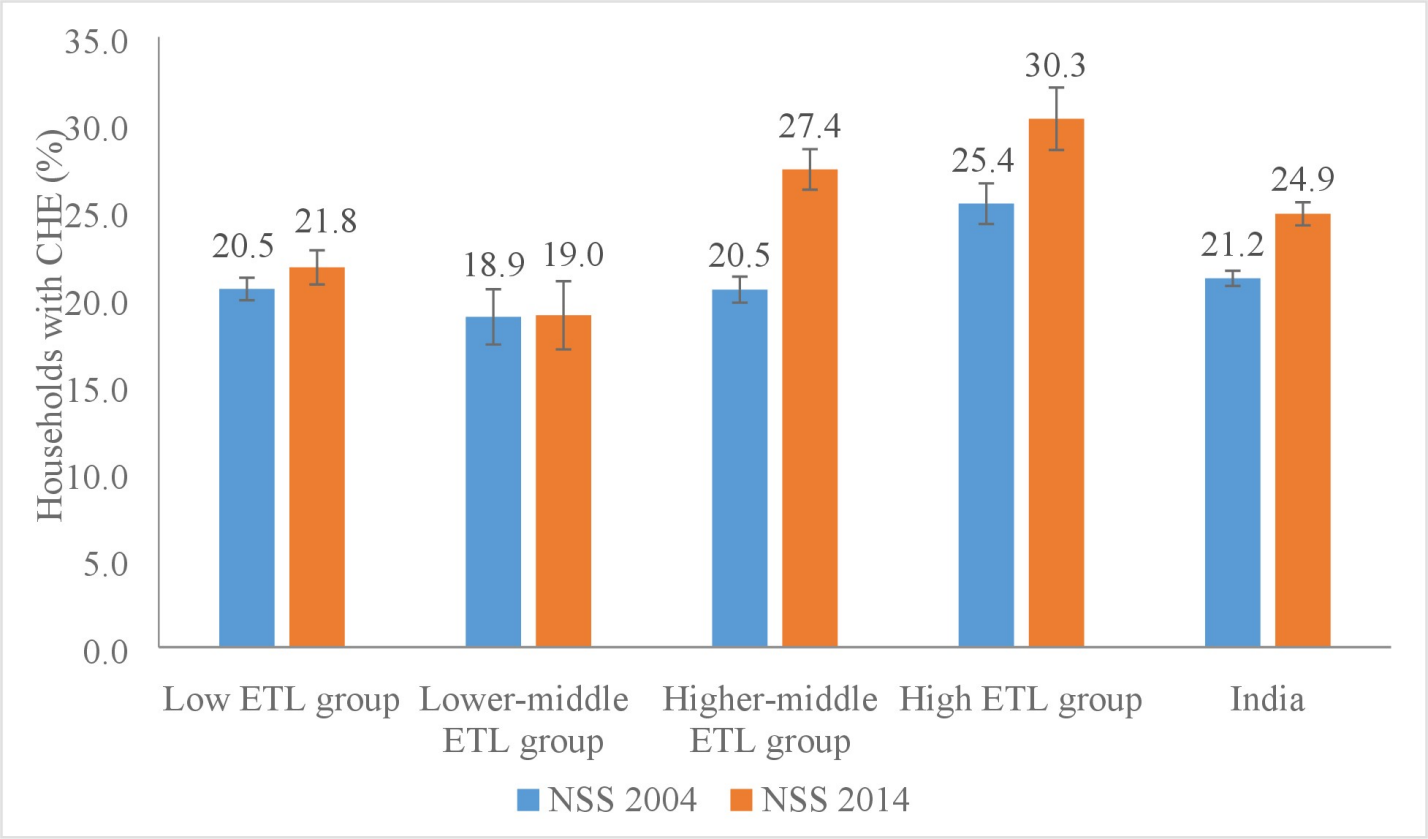
Percent of households with catastrophic health expenditure (CHE) with 95% CI by states in India grouped by epidemiological transition level (ETL), NSS 2004 and NSS 2014. NSS = National Sample Survey; CHE = Catastrophic health expenditure; CI = Confidence interval.

Overall, 24.9% of the households had CHE in 2014 which was 17.0% higher than that in 2004 (21.2%) (Fig 1). In 2014, CHE was higher in the high (30.3%) and higher-middle (27.4%) ETL state groups than the low (21.8%) and lower-middle (19.0%) groups. CHE increased between 2004 and 2014 in the higher-middle (33.6%) and high (19.1%) ETL state groups only (Fig 1).

**Fig 2:**
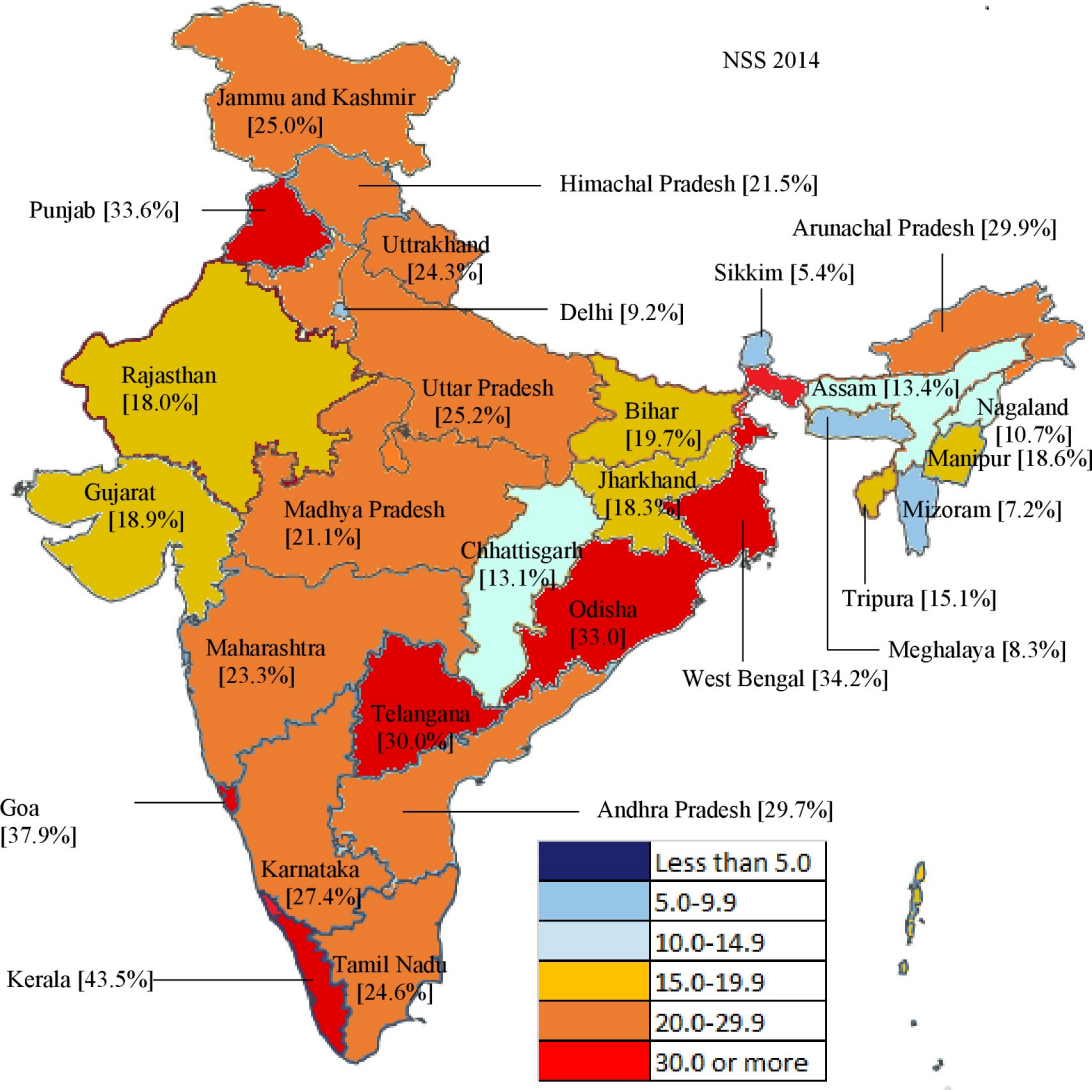
Heterogeneities in households with catastrophic health expenditure across states in India, NSS 2014. NSS = National Sample Survey.

**Fig 3:**
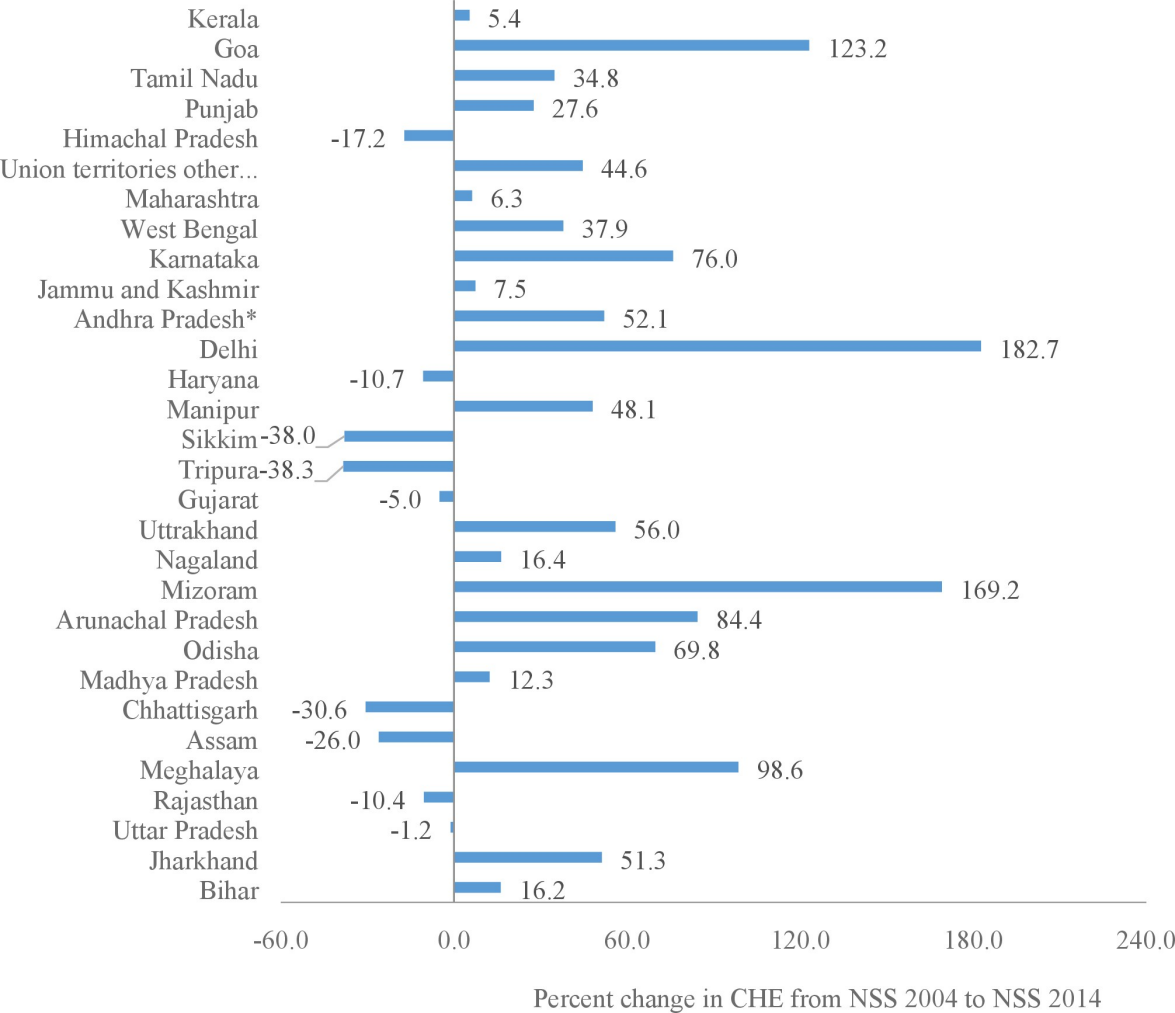
Percent change in catastrophic health expenditure (CHE) across states of India from NSS 2004 to NSS 2014. *For comparison purpose Andhra Pradesh has been considered as an undivided state in NSS 2014. NSS = National Sample Survey.

The proportion of households with CHE demonstrated enormous range; the highest CHE of an individual state in 2014 was 8.06-fold the lowest (Fig 2). Although CHE remained similar over time in the low and lower-middle ETL groups, some of the states within these groups also showed an increase in CHE (Fig 3). From 2004 to 2014, CHE increased significantly in the low ETL state of Odisha (69.8%) and Jharkhand (51.3%); lower-middle ETL states of Mizoram (169.2%), Arunachal Pradesh (84.4%) and Manipur (48.1%); higher-middle ETL state of Delhi (182.5%), Karnataka (76.0%), Andhra Pradesh (52.1%), and West Bengal (37.9%); and in the high ETL state of Tamil Nadu (34.8%) (Fig 3). CHE declined significantly in the north-east states of Assam (26.0%) in low ETL and Tripura (38.8%) in lower-middle ETL state group (Fig 3).

Overall, CHE was 1.26 times significantly higher among the rich than the poor in 2004; however, in 2014 this differential was not present because of the higher increase in CHE among the poor (35.9%) than rich (11.8%) (Table 2). Concentration index also showed that CHE was concentrated among the rich in 2004 (C: 0.053; 95% CI: 0.041, 0.065) and equally distributed among rich and poor in 2014 (Table 3). From 2004 to 2014, proportion of households with CHE increased significantly for the poor in the low (31.3%) and lower-middle (91.4%) ETL state group, and for the rich in the high ETL group (24.1%). However, in the higher-middle ETL group it increased substantially for the poor (46.0%), lower-middle (62.5%), middle (41.4%) and higher-middle (26.6%) quintiles (Table 2). CHE continued to be concentrated among the rich in high ETL group (C: 0.054; 95% CI: 0.020, 0.089 in 2014) however, in the other ETL groups CHE was generally concentered among the rich in 2004 and among the poor in 2014 (Table 3).

**Table 2:** Households with catastrophic health expenditure (CHE) across ETL state groups by MPCE quintiles in India, NSS 2004 and NSS 2014.

ETL state groups/MPCE quintiles	Households with CHE (95% CI)		Percent change in CHE from NSS 2004 to NSS 2014
India	NSS 2004	NSS 2014	
Poor	18.0 (17.1 to 18.9)	24.5 (23.0 to 26.0)	35.9
Lower-middle	19.0 (18.1 to 19.9)	24.4 (22.9 to 25.9)	28.6
Middle	22.7 (21.7 to 23.7)	24.7 (23.2 to 26.2)	8.9
Higher-middle	23.4 (22.4 to 24.4)	25.3 (23.8 to 26.8)	8.0
Rich	22.7 (21.7 to 23.7)	25.3 (23.9 to 26.7)	11.8
**Low ETL state group**			
Poor	17.4 (16.3 to 18.6)	22.9 (21.0 to 24.8)	31.3
Lower-middle	18.7 (17.4 to 20.0)	21.5 (19.4 to 23.5)	14.8
Middle	22.8 (21.3 to 24.3)	19.3 (17.3 to 21.2)	-15.6
Higher-middle	23.3 (21.6 to 24.9)	21.6 (19.2 to 24.1)	-7.1
Rich	23.1 (21.2 to 25.0)	24.1 (21.2 to 27.0)	4.3
**Lower-middle ETL state group**			
Poor	14.6 (10.6 to 18.7)	28.0 (19.8 to 36.2)	91.4
Lower-middle	20.9 (16.6 to 25.3)	20.6 (15.6 to 25.6)	-1.6
Middle	18.6 (14.7 to 22.5)	19.4 (15.0 to 23.7)	4.0
Higher-middle	19.0 (15.9 to 22.1)	16.2 (12.8 to 19.6)	-14.6
Rich	19.4 (16.6 to 22.2)	18.0 (14.5 to 21.6)	0.93
**Higher-middle ETL state group**			
Poor	19.1 (17.4 to 20.7)	27.8 (24.8 to 30.9)	46.0
Lower-middle	18.0 (16.4 to 19.6)	29.3 (26.5 to 32.1)	62.5
Middle	22.0 (20.3 to 23.6)	31.0 (28.2 to 33.9)	41.3
Higher-middle	22.3 (20.6 to 24.0)	28.2 (25.7 to 30.7)	26.6
Rich	20.9 (19.2 to 22.6)	22.0 (20.1 to 24.0)	5.4
**High ETL state group**			
Poor	19.1 (16.1 to 22.1)	27.7 (21.1 to 34.3)	44.9
Lower-middle	22.2 (19.5 to 24.9)	25.9 (21.0 to 30.8)	16.9
Middle	25.7 (22.7 to 28.6)	26.8 (22.7 to 30.8)	4.2
Higher-middle	28.2 (25.6 to 30.8)	30.2 (26.9 to 33.5)	7.1
Rich	27.5 (25.5 to 29.5)	34.1 (30.9 to 37.3)	24.1

ETL = Epidemiological transition level; NSS = National Sample Survey; MPCE = Monthly per capita consumption expenditure.

**Table 3:** Inequality in the burden of out-of-pocket health payments across states and ETL groups in India, NSS 2004 and NSS 2014.

State and ETL groups	Concentration index for CHE (95% CI)	
	NSS 2004	NSS 2014
**India**	**0.053 (0.041 to 0.065)**	**0.007 (-0.008 to 0.022)**
**Low ETL state group**	**0.067 (0.049 to 0.085)**	**-0.011 (-0.037 to 0.015)**
Bihar	0.048 (-0.003 to 0.100)	-0.056 (-0.137 to 0.024)
Chhattisgarh	-0.025 (-0.105 to 0.055)	0.101 (-0.056 to 0.258)
Jharkhand	0.107 (0.028 to 0.187)	0.073 (-0.064 to 0.210)
Madhya Pradesh	0.069 (0.016 to 0.122)	0.011 (-0.057 to 0.079)
Odisha	0.086 (0.032 to 0.139)	-0.005 (-0.066 to 0.056)
Rajasthan	0.065 (0.016 to 0.113)	0.026 (-0.049 to 0.100)
Uttar Pradesh	0.055 (0.027 to 0.084)	0.010 (-0.032 to 0.052)
Meghalaya	0.260 (0.132 to 0.389)	-0.052 (-0.257 to 0.154)
Assam	0.042 (-0.024 to 0.108)	-0.159 (-0.266 to -0.051)
**Lower-middle ETL state group**	**0.015 (-0.032 to 0.063)**	**-0.066 (-0.125 to -0.008)**
Uttrakhand	0.159 (0.040 to 0.278)	-0.166 (-0.300 to -0.033)
Arunachal Pradesh	-0.075 (-0.220 to 0.070)	-0.058 (-0.183 to 0.067)
Mizoram	0.032 (-0.230 to 0.294)	-0.050 (-0.304 to 0.203)
Nagaland	-0.088 (-0.365 to 0.188)	0.078 (-0.145 to 0.302)
Tripura	-0.037 (-0.110 to 0.036)	0.018 (-0.119 to 0.155)
Sikkim	-0.154 (-0.337 to 0.030)	0.106 (-0.067 to 0.279)
Manipur	0.285 (0.202 to 0.367)	-0.098 (-0.179 to -0.018)
Gujarat	0.014 (-0.044 to 0.072)	-0.036 (-0.108 to 0.035)
**Higher-middle ETL state group**	**0.032 (0.011 to 0.054)**	**-0.049 (-0.072 to -0.026)**
Haryana	0.007 (-0.061 to 0.076)	0.069 (-0.043 to 0.180)
Delhi	0.115 (-0.101 to 0.332)	-0.183 (-0.355 to -0.010)
Andhra Pradesh*	0.070 (0.019 to 0.121)	-0.020 (-0.071 to 0.030)
Jammu & Kashmir	-0.090 (-0.178 to -0.002)	-0.080 (-0.181 to 0.021)
Karnataka	0.033 (-0.022 to 0.088)	-0.067 (-0.127 to -0.008)
West Bengal	0.055 (0.018 to 0.091)	0.052 (0.010 to 0.094)
Maharashtra	0.048 (0.007 to 0.089)	-0.076 (-0.121 to -0.032)
Union Territories other than Delhi	-0.011 (-0.124 to 0.102)	-0.023 (-0.177 to 0.130)
**High ETL state group**	**0.052 (0.026 to 0.078)**	**0.054 (0.020 to 0.089)**
Himachal Pradesh	-0.033 (-0.096 to 0.030)	-0.137 (-0.254 to -0.020)
Punjab	0.079 (0.017 to 0.141)	-0.042 (-0.117 to 0.034)
Goa	0.060 (0.013 to 0.106)	0.082 (0.021 to 0.142)
Tamil Nadu	-0.023 (-0.309 to 0.263)	-0.031 (-0.235 to 0.173)
Kerala	-0.062 (-0.091 to -0.033)	-0.023 (-0.066 to 0.020)

ETL = Epidemiological transition level; NSS = National Sample Survey; CI = Confidence interval.

*For comparison purpose Andhra Pradesh has been considered as an undivided state in NSS 2014.

Inequality in incurring CHE varied across individual states in India. In 2004, Kerala (C: -0.062, 95% CI: -0.091 to -0.033) and Jammu and Kashmir (C: -0.090; 95% CI: -0.178 to -0.002) were the only states were CHE was disproportionately concentrated among the poor, whereas in 2014, West Bengal (C: 0.052, 95% CI: 0.010 to 0.094 in 2014) and Goa (C: 0.082; 95% CI: 0.021 to 0.142 in 2014) were the only states where CHE was disproportionately concentrated among the rich (Table 3). In 2004, CHE was generally higher among the rich, whereas, in 2014 it was generally distributed equally among the rich and poor. In 2014, states such as Assam, Uttrakhand, Manipur, Delhi, Karnataka, Maharashtra, and Himachal Pradesh had significant negative value of concentration index indicating the greater burden of OOP payments among the poor (Table 3).

## Discussion

The OOP payments for health care increased by 62% and the CHE increased by 17% from 2004 to 2014, indicating that the financial protection offered to patients by the health care system has remained inadequate in India. Behind this, however, are huge variations in the magnitude of OOP payments and CHE across the states of India which are at various levels of epidemiological transitions. In this study, we present the trends in OOP payments and CHE for states grouped by the level of epidemiological transition as well as the key findings for the individual states. We also provide evidence on the economic inequality in CHE across state groups for more focused attention on addressing these inequalities.

Four key findings emerge from this study. First, the high and higher-middle ETL state groups had higher CHE than the low and lower-middle group. Second, from 2004 to 2014, CHE increased for the higher-middle and high ETL state group only, but the states with substantial increase in CHE were distributed across all ETL groups. Third, the gap between the highest CHE of an individual state and the lowest was 8-fold in 2014. Fourth, CHE was concentrated among the rich in 2004 for most of India, but in 2014 CHE was distributed equally among rich and poor because of the substantial increase in CHE among the poor over time.

The disease epidemiology in India has changed towards the predominance of non-communicable diseases and injuries over the past two decades. All states in India have a higher burden of NCDs and injuries than CDs; however, the states vary considerably in their level of epidemiological transition [23]. The disease pattern has a direct link with the volume and type of health services needed which subsequently has an impact on the health expenditures [31–33]. Higher prevalence of NCDs in higher-middle and high ETL groups could be a contributor to the higher burden of CHE in these states. Evidence suggest that the treatment cost for NCDs is usually high in India because much of the care for NCDs is provided in the private sector [34–39]. However, NCDs would also be a contributor to CHE in the low ETL state groups because of their increasing burden in these states. Evidence on the burden of OOP payments by disease type would provide further information on the type and the quantum of services needed across states in India. The states at the higher level of epidemiological transition could also have higher CHE than other ETL state groups because of the higher utilisation of health care. The lower burden of CHE in the states at lower level of epidemiological transition is an important finding from the policy perspective as it likely reflects the unmet need for health care. Due to the lack of financial resources to pay for the high cost of health care, those in the lower ETL states, might not even be reaching the health facility. The changing disease epidemiology in India calls for strengthening the public facilities to provide comprehensive health care, particularly for the treatment of NCDs at low cost to provide financial protection.

Economic growth resulting in higher demand for expanded health care provisions, health service access and use are other important determinants resulting in catastrophic health expenditure [40]. States at the higher level of epidemiological transition are also economically well-off. A recent study used a composite index of socioeconomic development of states based on their per-capita income, average educational attainment and fertility rates, and found it to be inversely related to epidemiological transition ratio, with a correlation coefficient of -1.81 in 2016 [22]. The wealthier states spend more on health care, offer more extensive provision of private health care services [41–43], and their residents also have relatively more resources to invest in health [44] which often results in higher CHE in the absence of adequate risk pooling mechanism. Projection of CHE till 2040 in 179 countries found that if future expansion of health services availability is more rapid than the shift towards prepayments, CHE may increase with economic growth [44].

Our data showed substantial variation across states in the incidence of catastrophic health expenditure in the most recent health care utilisation survey ranging from less than 10% to more than 40%. Another study also found that the variation in CHE in India was particularly large at the state level than the regional and district levels, and the factors that drive CHE variability were specific to the state [45]. Given the contextual complexities, the state-specific approaches to health system in India would lead to better provision of services and financial risk protection than a national health system [46]. Our findings also support that one-size-fits-all approach in framing health policies would not be sufficient to meet the diverse health care needs of the states. For instance, the similar burden of CHE found in our study in the less developed state of Odisha, and in the economically advanced state of Punjab and West Bengal may be due to different reasons. In the developed states, high CHE could be a result of the greater awareness of health benefits, higher ability to pay and the preference for using private health facility over public health facility, but in the less developed states it could be due to low household income, lack of financial protection and unavailability of the public health infrastructure. A more detailed study of the contextual factors that may explain the variation in CHE across states is needed to better guide health policy framework in India.

We found that from 2004 to 2014, CHE increased substantially for some states spread across all ETL groups. The increase in CHE among states belonging to low, lower-middle, and higher-middle ETL groups was mainly due to the increased burden of OOP payments among the poor over time, whereas in the high ETL group it was rich who had higher incidence of CHE. The inequality in CHE was disproportionately higher among the rich in 2004 however, in 2014 CHE was either equally distributed among rich and poor or was pro-poor. This is against the equity considerations that demands that poorer households should not be disproportionately burdened with health expenses as compared to more affluent households [47]. Increasing usage of health services for the NCDs, higher utilisation of private facility due to the poor quality of health services and inadequate NCD service provision in public sector, and the lag in creating adequate provision for financial protection are plausible reasons for the increase in catastrophic health expenditure among poor in the states at lower level of epidemiological transition. Evidence suggest that Rashtriya Swasthaya Bima Yojana and other such state health insurance programmes have increased access of poor patients to hospitals, particularly private hospitals, but have not protected them against financial burden [35, 48–50]. Better provision of quality health care should be accompanied by financial protection measures to safeguard the poor from increasing CHE in India. To effectively reduce the incidence of CHE, government should focus on increasing the share of total health expenditure that is prepaid, particularly through taxes and mandatory contributions [5].

Creating provision for quality health care in public sector and providing financial protection from OOP payments are two sides of the UHC coin that should be undertaken simultaneously to realize the goal. The National Health Policy 2017 has included both these components by aiming to increase the utilisation of public health facility by 50% from the current levels, and reduce the proportion of households with CHE by 25%, by 2025 [8]. It also aims to increase the government health expenditure from 1.15% to 2.5% of GDP by 2025 [8]. Some studies have shown that funding UHC in India, would require increasing the government health expenditure up to 6% of gross domestic product (GDP) [51, 52]. India has taken the first move towards universal health care by launching Ayushman Bharat—National Health Protection Mission which aims to provide cost coverage, up to INR 500,000 (US$ 7,350) annually to over 100 million poor families for hospitalised secondary and tertiary care [53]. This is a significant improvement over the coverage of INR 30,000 (US$ 440) per family per year under the previous health insurance scheme Rashtriya Swasthaya Bima Yojana [54]. Another major initiative is the provision of creating 150,000 health and wellness centers to provide comprehensive primary and secondary health care including non-communicable diseases in order to strengthen the public health care system in India [8]. The state specific CHE trends from our study is a useful input for planning of the National Health Protection Mission such that it meets the requirements of each state, and achieve the goal of equity in health care.

## Supporting information

S1 Table Items used in household health care utilisation surveys to assess out-of-pocket payments (OOP) for outpatient and inpatient care, India. (DOCX)
