# Supplementary material for: Variations in catastrophic health expenditure across the states of India: 2004 to 2014
Source: PLoS One. 2018 Oct 22;13(10):e0205510. doi: 10.1371/journal.pone.0205510 (PMC6197636; doi:10.1371/journal.pone.0205510)
Supplement: S1 Table — (DOCX) [file pone.0205510.s001.docx]

**S1 Table**. **Items used in household health care utilisation surveys to assess out-of-pocket payments (OOP) for outpatient and inpatient care, India**

| **Survey** | **Type of care** | **Recall period** | **Recorded items paid OOP** |
| --- | --- | --- | --- |
| NSS 2004 | Outpatient | 15 days | Doctor's/surgeon's fee-hospital staff |
|  |  |  | Doctor's/surgeon's fee- other specialists |
|  |  |  | Medicines- from hospital |
|  |  |  | Medicines- from outside |
|  |  |  | Diagnostic tests |
|  |  |  | Attendant charges |
|  |  |  | Physiotherapy |
|  |  |  | Personal medical appliances |
|  |  |  | Food and other materials |
|  |  |  | Blood, oxygen cylinder |
|  |  |  | Services (e.g. ambulance) |
|  |  |  | Expenditure not elsewhere reported |
|  |  |  | Transport charges (other than ambulance) |
|  |  |  | Lodging charges of ailing person and escort(s) |
|  |  |  | Other expenses |
|  |  | 1 year | Vaccinations for children aged 0-4 years |
|  |  |  | Prenatal care |
|  |  |  | Childbirth (not in hospital) |
|  |  |  | Postnatal care |
|  | Inpatient care | 1 year | Doctor's/surgeon's fee-hospital staff |
|  |  |  | Doctor's/surgeon's fee- other specialists |
|  |  |  | Medicines- from hospital |
|  |  |  | Medicines- from outside |
|  |  |  | Diagnostic tests |
|  |  |  | Bed charges |
|  |  |  | Attendant charges |
|  |  |  | Physiotherapy |
|  |  |  | Personal medical appliances |
|  |  |  | Food and other materials |
|  |  |  | Blood, oxygen cylinder |
|  |  |  | Services (e.g. ambulance) |
|  |  |  | Expenditure not elsewhere reported |

(*continues*…)

(…*continued*)

| **Survey** | **Type of care** | **Recall period** | **Recorded items paid OOP** |
| --- | --- | --- | --- |
| NSS 2004 | Inpatient | 1 year | Transport (other than ambulance) |
|  |  |  | Lodging charges of escort(s) |
|  |  |  | Other expenses |
|  |  |  | Child birth |
| NSS 2014 | Outpatient | 15 days | Doctor’s/ surgeon’s fee (hospital staff/ other specialists) |
|  |  |  | Medicines: AYUSH^*^ |
|  |  |  | Medicines: other than AYUSH^*^ |
|  |  |  | Diagnostic tests |
|  |  |  | Other medical expenses (attendant charges, physio-therapy, personal medical appliances, blood, oxygen, etc.) |
|  |  |  | Transport for patient |
|  |  |  | Other expenses (food, transport for others, expenditure on escort, etc.) |
|  |  | 1 year | Prenatal care |
|  |  |  | Postnatal care |
|  | Inpatient | 1 year | Package components |
|  |  |  | Doctor’s/ surgeon’s fee (hospital staff/ other specialists) |
|  |  |  | Medicines |
|  |  |  | Diagnostic tests |
|  |  |  | Bed charges |
|  |  |  | Other medical expenses (attendant charges, physiotherapy, personal medical appliances, blood, oxygen, etc.) |
|  |  |  | Transport for patient |
|  |  |  | Other non-medical expenses (food, transport for others, expenditure on escort, lodging charges if any, etc.) |
|  |  |  | Child birth |

^*^Ayurveda, Yoga and Naturopathy, Unani, Siddha and Homoeopathy; NSS= National sample survey.
